# Regulatory mechanisms for the production of BAFF and IL-6 are impaired in monocytes of patients of primary Sjögren's syndrome

**DOI:** 10.1186/ar3493

**Published:** 2011-10-21

**Authors:** Keiko Yoshimoto, Maiko Tanaka, Masako Kojima, Yumiko Setoyama, Hideto Kameda, Katsuya Suzuki, Kensei Tsuzaka, Yoko Ogawa, Kazuo Tsubota, Tohru Abe, Tsutomu Takeuchi

**Affiliations:** 1 Division of Rheumatology and Clinical Immunology, Department of Internal Medicine, Keio University School of Medicine, 35 Shinanomachi, Shinjuku, Tokyo 160-8582, Japan; 2 Division of Rheumatology and Clinical Immunology, Saitama Medical Center, Saitama Medical University, 1981 Kamodatsujido-cho, Kawagoe, Saitama 350-8550, Japan; 3 Department of Internal Medicine, Tokyo Dental College Ichikawa General Hospital, 5-11-3 Sugano, Ichikawa, Chiba 272-8513, Japan; 4 Department of Ophthalmology, Keio University School of Medicine, 35 Shinanomachi, Shinjuku, Tokyo 160-8582, Japan

## Abstract

**Introduction:**

In this study, we investigated possible aberrations of monocytes from patients with primary Sjögren's syndrome (pSS). We focused on B-cell-activating factor of the TNF family (BAFF) and IL-6 because they are both produced by monocytes and are known to be involved in the pathogenesis of pSS.

**Methods:**

Peripheral monocytes were prepared from both pSS patients and normal individuals. The cells were stimulated *in vitro *with IFN-γ, and the amounts of IL-6 and soluble BAFF (sBAFF) produced by the cells were quantitated. The effect of sBAFF itself on the production of IL-6 was also studied. To investigate the response of pSS monocytes to these stimuli, the expression levels of the genes encoding BAFF receptors and IL-6-regulating transcription factors were quantitated.

**Results:**

Peripheral pSS monocytes produced significantly higher amounts of sBAFF and IL-6 than normal monocytes did, even in the absence of stimulation. The production of these cytokines was significantly increased upon stimulation with IFN-γ. The elevated production of IL-6 was significantly suppressed by an anti-BAFF antibody. In addition, stimulation of pSS monocytes with sBAFF induced a significant increase in IL-6 production. Moreover, the expression levels of a BAFF receptor and transcription factors regulating IL-6 were significantly elevated in pSS monocytes compared to normal monocytes.

**Conclusions:**

The results of the present study suggest that the mechanisms underlying the production of sBAFF and IL-6 are impaired in pSS monocytes. Our research implies that this impairment is due to abnormally overexpressed IL-6-regulating transcription factors and a BAFF receptor. These abnormalities may cause the development of pSS.

## Introduction

Sjögren's syndrome (SS) is an autoimmune disease which primarily affects the salivary and lachrymal glands. Major clinical manifestations of primary SS (pSS) are xerostomia and keratoconjunctive sicca, which are consequences of lesions of the salivary glands and lachrymal glands, respectively. Accumulating evidence suggests that lymphocytic infiltrate of exocrine glands plays a key role in lesion formation and the subsequent dysfunction of the glands [1].

B-cell-activating factor of the TNF family (BAFF) (tumor necrosis factor ligand superfamily, member 13b) is a cytokine which is primarily produced by monocytes and dendritic cells [2-4] in addition to T cells [5,6]. It plays a crucial role in the proliferation, differentiation and survival of B cells [2,4,5,7]. BAFF is a type II membrane-bound protein of 285 amino acid residues. A C-terminal fragment of 152 amino acid residues is released from cells as soluble BAFF (sBAFF) [5]. sBAFF binds to its receptors (that is, transmembrane activator and calcium modulator and cyclophilin ligand interactor (TACI), B cell maturation antigen (BCMA) and B cell activating factor receptor (BAFF-R) [8-14]), possibly as a trimer [8,11,13], and elicits signal transduction through several pathways [10,11,13,15,16]. It is noteworthy that transgenic mice that overexpress BAFF in lymphoid cells develop hyperplasia of mature B cells [8,17,18] or pSS-like pathology [19]. BAFF is also elevated in the serum of pSS patients [20,21] and strongly expressed in the lymphocytes infiltrating the salivary glands [22,23]. Moreover, elevated production of BAFF has been linked to the development of another autoimmune disease, systemic lupus erythematosus [24-26].

Notably, systemic and/or local concentrations of several other cytokines, such as IL-6, are also significantly elevated in pSS patients compared to normal individuals [27,28]. IL-6 promotes the differentiation of B cells [29], which play a pivotal role in the production of autoantibodies and hence in the development of pSS. Since monocytes produce both IL-6 [30] and BAFF [2,4,31], we hypothesized that the production of these cytokines is dysregulated in pSS monocytes. If that is the case, aberrations of pSS monocytes may be implicated in the abnormal production of autoreactive immunoglobulin G (IgG) by B cells, which is involved in the pathogenesis of autoimmune diseases such as pSS [32]. In the present study, we demonstrate that the regulatory mechanisms for the production of these cytokines are impaired in pSS monocytes.

## Materials and methods

### Patients and controls

Venous blood samples were collected from pSS patients (*n *= 13 females ages 32 to 64 years (average age = 50.5)) and normal individuals (*n *= 12 females ages 26 to 60 years (average age = 43.5)) after receiving their informed consent. Patients fulfilled the American-European Consensus Group criteria for pSS [33]. At the time of the collection of blood samples, two patients (15.4%) were receiving prednisolone at a daily dose < 5 mg. The remaining patients were free of medication. This study was approved by the ethics committees at Keio University School of Medicine and Saitama Medical University.

### Stimulation of peripheral monocytes *in vitro*

Peripheral monocytes were isolated as follows: Whole blood was mixed with RosetteSep Human Monocyte Enrichment Cocktail (StemCell Technologies, Vancouver, BC, Canada) and centrifuged over Ficoll-Hypaque (Beckman Coulter, Fullerton, CA, USA). A monocyte-enriched fraction was collected and cultured overnight in RPMI 1640 (American Tissue Culture Collection, Manassas, VA, USA) supplemented with 10% FCS in a humidified incubator (7% CO_2_) at 37°C so that the expression of various stress-induced genes subsided. The cells were then washed once with the medium to remove debris. Fluorescence-activated cell sorting (FACS) analysis of the cells demonstrated that > 96% of the living cells were CD14-positive.

The monocytes were cultured in the absence or presence of various concentrations of IFN-γ or sBAFF, and the cumulative production of sBAFF and/or IL-6 was examined by ELISA. The production was dependent on the incubation period. The optimal incubation period was found to be 96 hours. The production of the cytokines increased almost in proportion to the concentration of stimuli up to 200 ng/ml IFN-γ or 2 μg/ml sBAFF.

### Antibodies and recombinant proteins

An anti-BAFF mAb for ELISA was prepared in our laboratory [6]. A rabbit polyclonal anti-BAFF antibody and recombinant human sBAFF were purchased from Chemicon International (Temecula, CA, USA). Recombinant human IFN-γ, a control mouse IgG1, and mAbs for measurement of the amount of IL-6 by ELISA (MQ2-13A5 and MQ2-39C3 for capture and detection, respectively) and for FACS analysis (CD4-APC (RPA-T4) for T cells, CD14-PE-Cy7 (M5E2) for monocytes, CD20-APC-Cy7 (L27) for B cells and CD268-FITC (11C1) for BAFF-R) were purchased from BD Biosciences/Pharmingen (San Diego, CA, USA). An anti-TACI antibody for FACS analysis (CD267-PE (FAB1741P)) was purchased from R&D Systems (Minneapolis, MN, USA).

### ELISA

Monocytes were cultured at 2.5 × 10^5^/ml for 96 hours in a 24-well plate (2 ml/well) in the presence of stimuli (that is, recombinant human IFN-γ or recombinant human sBAFF). The amounts of sBAFF (in response to IFN-γ as a stimulus) and IL-6 (in response to IFN-γ or sBAFF as stimuli) in the culture supernatants were measured by sandwich ELISA according to previously described methods [6], except for the concentrations of capture and detection antibodies for IL-6, which were prepared at 0.5 μg/ml.

For quantitation of sBAFF, we used our own anti-BAFF mAb, which specifically detects sBAFF and does not react with a proliferation-inducing ligand (APRIL) [6]. In our hands, the sensitivity of our ELISA system was better than that of commercially available ELISA kits (R&D Systems) in the range of 0.4 to 100 ng/ml sBAFF (data not shown).

### Quantitation of the gene expression levels

The expression levels of BAFF, BAFF-R, TACI, NF-IL6, NF-IL6β, NF-κB1, NF-κB2 and glyceraldehyde 3-phosphate dehydrogenase (GAPDH) were quantitated by using a method described previously [6]. The following oligonucleotides were used as primers for PCR: 5'-ggaatctctgatgccacagctc and 5'-accttcaagggctgtcaaagatg (BAFF-R); 5'-agcatcctgagtaatgagtggcc and 5'-gagcttgttctcacagaagtatgc (TACI); 5'-aaaactttggcactggggcacttg and 5'-catctttaagcgattactcagggc (NF-IL6); 5'-agatgcagcagaagttggtggag and 5'-tagcttctctcgcagtttagtgg (NF-IL6β); 5'-atgggatctgcactgtaactgc and 5'-tcatagatggcgtctgataccacg (NF-κB1); 5'-cctgactttgagggactgtatcca and 5'-gcagcatttagcagcaaggtcttc (NF-κB2). Primer sets for BAFF and GAPDH were designed as described previously [6]. The expression level of each gene underwent dual normalization against GAPDH expression and expression of the same gene in unstimulated normal monocytes.

### FACS analysis

FACS and data analyses were carried out on a MACSQuant Analyzer (Miltenyi Biotec, Bergisch Gladbach, Germany). FACS analysis of cells in whole blood was carried out according to methods recommended by the manufacturer of the antibodies (BD Biosciences/Pharmingen).

### Statistical analysis

Differences between groups were examined for statistical significance by using the two-tailed Student's *t*-test for single comparisons. Two-way analysis of variance (ANOVA) was also employed when appropriate. A *P *value less than 0.05 denoted the presence of a statistically significant difference.

## Results

### Aberrant production of sBAFF by pSS monocytes

**Figure 1:**
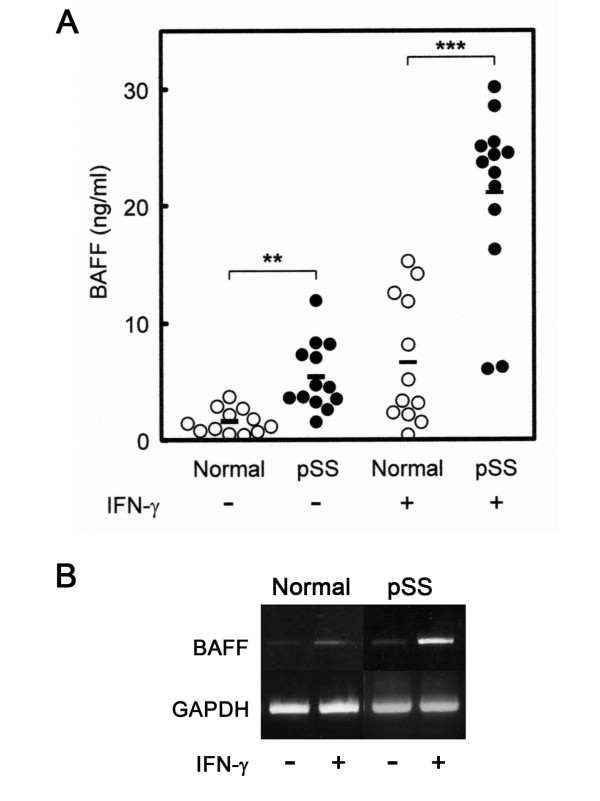
**Production of sBAFF by peripheral monocytes**. Monocytes (2.5 × 10^5^/ml) were cultured for 96 hours in the absence (-) or presence (+) of recombinant human IFN-γ (200 ng/ml). **(A) **The amounts of soluble B-cell-activating factor of the TNF family (sBAFF) in the culture supernatants of normal individuals (open circles) and primary Sjögren's syndrome (pSS) patients (closed circles) were quantitated in triplicate measurements by sandwich ELISA. Bars indicate means. **P < 0.01 and ***P < 0.001. **(B) **Total RNAs were prepared from normal and pSS monocytes and subjected to RT-PCR for BAFF and glyceraldehyde 3-phosphate dehydrogenase (GAPDH). Representative data are shown.

Peripheral monocytes were prepared from primary pSS patients and normal individuals. The clinical characteristics of the pSS patients involved in this study are listed in Table 1. The cells were cultured for 96 hours in the absence or presence of IFN-γ (200 ng/ml), which is known to activate monocytes [34] and upregulate the expression of BAFF [2]. Stimulation of the cells was confirmed by the induction of interferon-gamma inducible protein 10 (data not shown). pSS monocytes released a significantly higher amount of sBAFF (5.4 ± 0.8 ng/ml) into the culture media than normal monocytes did (1.6 ± 0.3 ng/ml), even in the absence of stimulation, suggesting dysregulated production of sBAFF in pSS monocytes (Figure 1A, "Normal -" and "pSS -"). IFN-γ stimulation (Figure 1A, "Normal +" and "pSS +") resulted in an increase in sBAFF in both normal (6.6 ± 1.6 ng/ml) and pSS monocytes (21.1 ± 2.1 ng/ml).

**Table 1:** Clinical characteristics of primary Sjögren's syndrome patients involved in this study

Patient characteristics	Clinical data
Female (%)	100
Mean age ± SD (years)	50.5 ± 10.2
Subjective ocular dryness (%)	100
Subjective oral dryness (%)	100
Presence of anti-SSA/Ro (%)	61.5
Presence of anti-SSB/La (%)	23.1
Presence of rheumatoid factor (%)	53.8
Mean serum IgG ± SD (μg/ml)	1,979.6 ± 870.5
Steroid medication (%)	15.4

IgG = immunoglobulin G.

RT-PCR analysis indicated that the expression of the *BAFF *gene in pSS monocytes was distinctly elevated upon stimulation with IFN-γ (Figure 1B). Quantitative RT-PCR analysis indicated that the relative expression level of the *BAFF *gene was about three times higher in pSS monocytes than in normal monocytes under unstimulated conditions. The expression levels increased about sixfold in both normal and pSS monocytes upon stimulation with IFN-γ. These data are basically consistent with the results derived by ELISA. Therefore, we postulated that the elevated production of sBAFF was the consequence of the enhanced expression of the *BAFF *gene.

### Aberrant production of IL-6 by pSS monocytes

**Figure 2:**
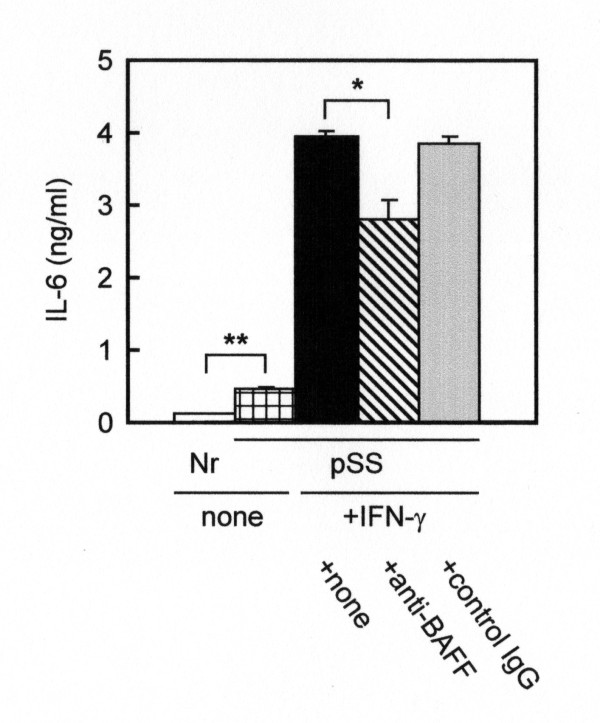
**Production of IL-6 by peripheral monocytes stimulated with IFN-γ**. Monocytes (2.5 × 10^5^/ml) prepared from normal individuals (Nr) (open column) and primary Sjögren's syndrome (pSS) patients (checkerboard column) were cultured for 96 hours without stimulation. pSS monocytes were similarly cultured in the presence of 200 ng/ml of recombinant human IFN-γ (closed, hatched and gray columns), and simultaneously exposed to none (closed column), an anti-BAFF antibody (10 μg/ml; hatched column) or a control IgG (10 μg/ml; gray column). The amounts of IL-6 in the culture supernatants were measured by sandwich ELISA. BAFF = B-cell-activating factor of the TNF family; IgG = immunoglobulin G. Data represent means ± SEM. *P < 0.05 and **P < 0.01.

We also investigated whether the production of IL-6 by pSS monocytes was abnormal. As indicated in Figure 2, pSS monocytes produced significantly higher amounts of IL-6 than normal monocytes without stimulation (Figure 2, open column vs checkerboard column; *P *< 0.01). Stimulation of pSS monocytes with 200 ng/ml IFN-γ induced a striking increase (8.6-fold; *P *< 0.001) in IL-6 production (Figure 2, checkerboard column vs closed column). Since IFN-γ induced the expression of BAFF (Figure 1) and BAFF is able to activate monocytes [35,36], these results suggest that BAFF produced by monocytes may act in an autocrine fashion to augment the expression of IL-6. To test this hypothesis, we stimulated pSS monocytes with IFN-γ in the presence of an anti-BAFF mAb [6]. Interestingly, the mAb suppressed IL-6 production in part, but significantly so (*P *< 0.05) (Figure 2, closed column vs hatched column, whereas a control antibody had no effect (Figure 2, closed column vs gray column). These results suggest that the signal transduction pathway mediated by BAFF is implicated in the regulation of IL-6 production by IFN-γ-primed monocytes.

**Figure 3:**
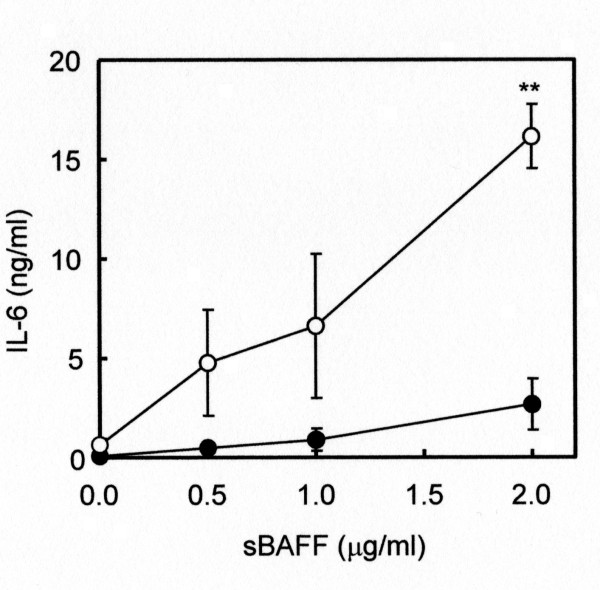
**Production of IL-6 by peripheral monocytes stimulated with sBAFF**. Monocytes (2.5 × 10^5^/ml) prepared from normal individuals (closed circles) and primary Sjögren's syndrome (pSS) patients (open circles) were cultured for 96 hours in the presence of 0, 0.5, 1.0 and 2.0 μg/ml of recombinant human soluble B-cell-activating factor of the TNF family (sBAFF). The amounts of IL-6 in the culture supernatants were measured by sandwich ELISA. Data represent means ± SEM. **P < 0.01.

If this is really the case, then exogenously supplemented sBAFF should affect the production of IL-6 by monocytes. As expected, recombinant human sBAFF induced the production of IL-6 by both normal (Figure 3, closed circles) and pSS (Figure 3, open circles) monocytes in a dose-dependent manner. pSS monocytes produced approximately six times more abundant IL-6 than normal monocytes in the presence of 2 μg/ml sBAFF (Figure 3). It should be noted that two-way ANOVA revealed that disease status (normal or pSS) had significantly stronger effects than stimulation with sBAFF (*P *< 0.001 for cell type × stimulation interaction) on IL-6 production by monocytes.

A 2,3-bis-(2-methoxy-4-nitro-5-sulfophenyl)-2*H*-tetrazolium-5-carboxanilide (XTT) assay in a separate experiment indicated that 2 μg/ml sBAFF supported the survival of both normal and pSS monocytes during the culture period. However, there was no significant difference in survival rates between normal and pSS monocytes (data not shown), suggesting that higher production of IL-6 was not simply a consequence of enhanced survival of monocytes. These data suggest that the regulatory mechanism for IL-6 production is aberrant in pSS monocytes.

### Aberrant expression of BAFF receptors in pSS monocytes

**Figure 4:**
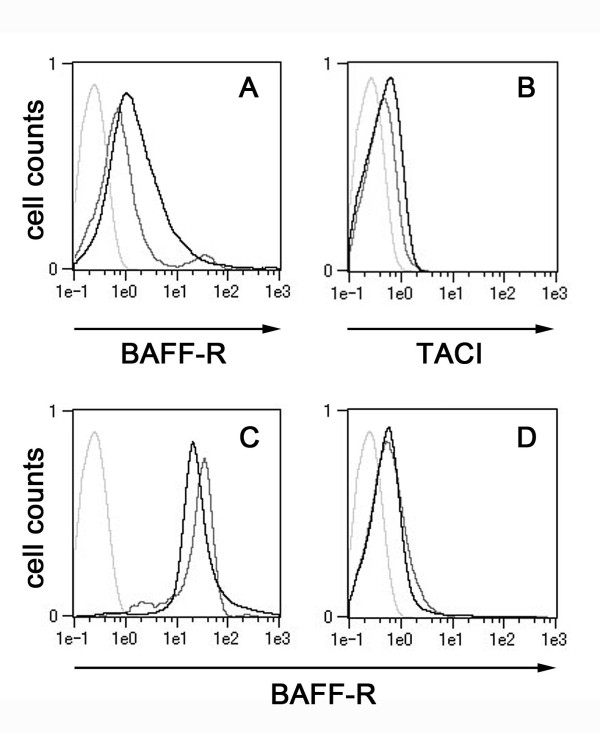
**Fluorescence-activated cell sorting analysis of monocytes and lymphocytes**. **(A) **and **(B) **Normal (gray line) and primary Sjögren's syndrome (pSS) (black line) monocytes were cultured for 96 hours without stimulation, and CD14^+^/BAFF-R^+ ^cells **(A) **and CD14^+^/TACI^+ ^cells **(B) **were examined by fluorescence-activated cell sorting (FACS) analysis. **(C) **and **(D) **Lymphocytes in whole blood samples of a normal individual (gray line) and a pSS patient (black line) were examined by FACS analysis for CD20^+^/BAFF-R^+ ^cells (B cells in part **(C)**) and CD4^+^/BAFF-R^+ ^cells (T cells in part **(D)**). Light gray lines represent isotype controls. Representative data derived by FACS analysis are shown. BAFF-R = B cell activating factor receptor; TACI = transmembrane activator and calcium modulator and cyclophilin ligand interactor.

Although it has been reported that BAFF receptors are mainly expressed in lymphocytes [37], our results suggest that BAFF receptors are also expressed in monocytes. RT-PCR detected mRNA for BAFF-R in monocytes. Notably, the expression level of BAFF-R was significantly elevated in pSS monocytes (2.1-fold; *P *< 0.001) compared to normal monocytes (Table 2). In accordance with these data, FACS analysis indicated that approximately 60% of pSS monocytes were BAFF-R-positive (mean fluorescence intensity (MFI) = 50), whereas only about 25% of normal monocytes were positive to the same antibody (MFI = 20) (Figure 4A). The proportion of TACI-positive monocytes was relatively low compared to BAFF-R-positive cells (Figure 4B). Although the population of TACI-positive monocytes seemed to increase slightly in pSS compared to control monocytes (Figure 4B), the expression level of the *TACI *gene was not significantly increased (Table 2).

**Table 2:** Expression of BAFF receptors in peripheral monocytes

Receptor	Normal	pSS
TACI	100.0 ± 17.6	121.7 ± 20.4
BAFF-R	100.0 ± 5.9	213.8 ± 14.9***

BAFF = B-cell-activating factor of the TNF family; pSS = primary Sjögren's syndrome; TACI = transmembrane activator and calcium-modulator and cyclophilin ligand interactor. Monocytes (2.5 × 10^5^/ml) prepared from normal individuals ("Normal") and pSS patients ("pSS") were cultured for 96 hours without stimulation. Total RNAs were extracted from the cells, and the expression levels of TACI and BAFF-R were quantitated. The relative expression levels of the genes are indicated. Data represent means ± SEM. Asterisk denotes statistically significant difference between "Normal" and "pSS." ***P < 0.001.

FACS analysis of lymphocytes in whole blood indicated that there were no significant differences between pSS patients and normal individuals in the population of BAFF-R-positive B and T cells (Figures 4C and 4D). All these data suggest that the expression of BAFF-R is dysregulated in pSS monocytes and that this dysregulation seems to be specific to monocytes among the cells examined thus far.

### Aberrant expression of transcription factors in pSS monocytes

In an attempt to elucidate a possible mechanism of sBAFF-mediated overproduction of IL-6 by pSS monocytes, we investigated the expression levels of transcription factors involved in the expression of the IL-6 gene (that is, NF-IL6 (CCAAT/enhancer-binding protein β), NF-IL6β (CCAAT/enhancer-binding protein δ), NF-κB1 and NF-κB2). The relative expression levels of all the transcription factors were significantly elevated in pSS monocytes compared with the control (Table 3). Remarkably, the relative expression level of NF-IL6 was more than six times higher in pSS monocytes than in normal monocytes. These data indicate that the expression of IL-6-regulating transcription factors was abnormally upregulated in pSS monocytes.

**Table 3:** Expression of transcription factors in peripheral monocytes

Transcription factor	Normal	pSS
NF-IL6	100.0 ± 16.0	623.6 ± 85.8***
NF-IL6β	100.0 ± 18.5	252.8 ± 51.5*
NF-κB1	100.0 ± 11.4	167.5 ± 23.4*
NF-κB2	100.0 ± 14.9	342.6 ± 45.4***

pSS = primary Sjögren's syndrome. Total RNAs were extracted from monocytes prepared as described in the Table 2 footnote, and the expression levels of NF-IL6, NF-IL6β, NF-κB1 and NF-κB2 were quantitated. The relative expression levels of the genes are indicated. Data represent means ± SEM. Asterisk denotes statistically significant difference between "Normal" and "pSS." *P < 0.05 and ***P < 0.001.

## Discussion

Several lines of circumstantial evidence have suggested that BAFF and IL-6 are implicated in the development of primary pSS [19-23,27,28,38]. In addition, these cytokines are produced by monocytes [2,4,39,40]. These findings prompted us to investigate the possibility of aberrations in the monocytes of pSS patients. We hypothesized that the production of these cytokines is dysregulated in pSS monocytes. To address this issue, we examined the production of these cytokines by peripheral pSS monocytes *in vitro *in response to IFN-γ, a cytokine known to upregulate BAFF expression [2,41]. As expected, pSS monocytes produced a higher amount of sBAFF than normal monocytes, even in the absence of stimulation (Figure 1A).

IFN-γ also induced the production of IL-6 by pSS monocytes. Interestingly, the induction was suppressed in part, but significantly, by an anti-BAFF antibody (Figure 2). In addition, exogenously supplemented sBAFF induced a striking increase in the production of IL-6 by pSS monocytes (Figure 3), whereas exogenously supplemented IL-6 had no effects on the production of sBAFF by the cells (data not shown). These data, together with the results shown in Figure 1A, collectively imply that BAFF produced by monocytes act in an autocrine fashion and that signal transduction pathways mediated by BAFF are likely involved in the regulation of IL-6 production by monocytes. Notably, two-way ANOVA indicated that pSS monocytes were more susceptible than normal monocytes to stimulation by sBAFF. This increased susceptibility may be due to an exaggeration of signals in pSS monocytes triggered by sBAFF.

BAFF is known to bind to several receptors, such as TACI, BAFF-R and BCMA [8,10,11,13]. BAFF binds TACI [42] and BAFF-R [43,44] with high affinity, whereas the binding affinity of BAFF to BCMA is very low [44,45]. We found that a relatively small population of normal monocytes was TACI-positive (Figure 4B) and that the expression level of TACI did not increase in pSS patients (Table 2). Interestingly, expression of BAFF-R, a BAFF-specific receptor, was significantly elevated in pSS monocytes compared to the control (Table 2). FACS analysis suggested that this elevation may be the consequence of an increase not only in the population of BAFF-R-positive cells but also in the expression of the *BAFF-R *gene in individual pSS monocytes (Figure 4A). Considering all of this information together, we believe that abnormally overexpressed BAFF-R may have contributed to the enhanced production of IL-6 by pSS monocytes upon stimulation with sBAFF (Figure 3). The increase in the population of BAFF-R-positive cells was specific to pSS monocytes among the cells examined thus far, and no significant differences were observed in the population of BAFF-R-positive lymphocytes between pSS and the normal control (Figures 4C and 4D).

To shed light on the aberrant production of IL-6 by pSS monocytes, we examined the expression levels of several transcription factors involved in the expression of IL-6. Interestingly, the expression levels of all the transcription factors examined in the present study were significantly elevated compared to normal monocytes (Table 3). The expression of these transcription factors was generally constitutive and insensitive to stimulation, in particular with regard to sBAFF (data not shown). The expression level of NF-IL6 was especially high among the transcription factors examined. The higher expression of these factors may have amplified a signal triggered by sBAFF which resulted in overproduction of IL-6 by pSS monocytes. On the basis of the results shown in Figure 2 andTable 3 we suppose that IFN-γ induces the production of IL-6 in pSS monocytes through at least two distinct pathways: one is direct activation of the IL-6 gene and the other is indirect activation of the gene mediated by sBAFF.

The relationship between the aberration of pSS monocytes and the clinical manifestations of the disease remains unclear. There was no significant correlation between the presence of rheumatoid factor, anti-SSA/Ro or anti-SSB/La in pSS patients and the amounts of IL-6 and sBAFF produced by pSS monocytes. However, dendritic cells have been observed in the salivary glands of pSS patients [46-48], and peripheral monocytes can migrate to the salivary glands and develop into dendritic cells [49-51]. In addition, the local concentration of IFN-γ in the salivary glands of pSS patients seems to be increased because of T cells' infiltrating the tissue [51,52]. Therefore, we hypothesize that monocyte-derived dendritic cells infiltrating the salivary glands of pSS patients are stimulated by IFN-γ to produce excessive amounts of BAFF and IL-6.

## Conclusions

Although the number of the patients involved in the current study was small, the data strongly suggest that monocytes of pSS patients are abnormally activated. We hypothesize that stimulation of pSS monocytes by IFN-γ is partly mediated by BAFF as a result of the abnormal overexpression of BAFF-R and that the signals are amplified by abnormal overexpression of transcription factors that regulate IL-6 production. We speculate that these aberrations may underlie the pathogenesis of pSS.

## Abbreviations

ANOVA: analysis of variance; ELISA: enzyme-linked immunosorbent assay; FCS: fetal calf serum; IFN: interferon; IL: interleukin; mAb: monoclonal antibody; MFI: mean fluorescence intensity; PCR: polymerase chain reaction; RT: reverse transcriptase; pSS: primary Sjögren's syndrome; sBAFF: soluble BAFF; TNF: tumor necrosis factor.

## Competing interests

The authors declare that they have no competing interests.

## Authors' contributions

KY and TT were responsible for the study design; the acquisition, analysis and interpretation of data; and manuscript preparation. MT, YS and MK contributed to the acquisition, analysis and interpretation of data. HK, KS, KeT, YO and KaT participated in the enrollment of patients into the study and assisted in the acquisition and interpretation of data. TA was involved in data interpretation and manuscript preparation. All authors read and approved the final manuscript for publication.

## References

[B1] Masaki Y Sugai S Lymphoproliferative disorders in Sjögren's syndrome Autoimmun Rev 2004 3 175 182 10.1016/S1568-9972(03)00102-2 15110228

[B2] Moore PA Belvedere O Orr A Pieri K LaFleur DW Feng P Soppet D Charters M Gentz R Parmelee D Li Y Galperina O Giri J Roschke V Nardelli B Carrell J Sosnovtseva S Greenfield W Ruben SM Olsen HS Fikes J Hilbert DM BLyS: member of the tumor necrosis factor family and B lymphocyte stimulator Science 1999 285 260 263 10.1126/science.285.5425.260 10398604

[B3] Shu HB Hu WH Johnson H TALL-1 is a novel member of the TNF family that is down-regulated by mitogens J Leukoc Biol 1999 65 680 683 10331498

[B4] Tribouley C Wallroth M Chan V Paliard X Fang E Lamson G Pot D Escobedo J Williams LT Characterization of a new member of the TNF family expressed on antigen presenting cells Biol Chem 1999 380 1443 1447 10.1515/BC.1999.186 10661873

[B5] Schneider P Mackay F Steiner V Hofmann K Bodmer JL Holler N Ambrose C Lawton P Bixler S Acha-Orbea H Valmori D Romero P Werner-Favre C Zubler RH Browning JL Tschopp J BAFF, a novel ligand of the tumor necrosis factor family, stimulates B cell growth J Exp Med 1999 189 1747 1756 10.1084/jem.189.11.1747 10359578 PMC2193079

[B6] Yoshimoto K Takahashi Y Ogasawara M Setoyama Y Suzuki K Tsuzaka K Abe T Takeuchi T Aberrant expression of BAFF in T cells of systemic lupus erythematosus, which is recapitulated by a human T cell line, Loucy Int Immunol 2006 18 1189 1196 10.1093/intimm/dxl053 16740602

[B7] Schiemann B Gommerman JL Vora K Cachero TG Shulga-Morskaya S Dobles M Frew E Scott ML An essential role for BAFF in the normal development of B cells through a BCMA-independent pathway Science 2001 293 2111 2114 10.1126/science.1061964 11509691

[B8] Gross JA Johnston J Mudri S Enselman R Dillon SR Madden K Xu W Parrish-Novak J Foster D Lofton-Day C Moore M Littau A Grossman A Haugen H Foley K Blumberg H Harrison K Kindsvogel W Clegg CH TACI and BCMA are receptors for a TNF homologue implicated in B-cell autoimmune disease Nature 2000 404 995 999 10.1038/35010115 10801128

[B9] Marsters SA Yan M Pitti RM Haas PE Dixit VM Ashkenazi A Interaction of the TNF homologues BLyS and APRIL with the TNF receptor homologues BCMA and TACI Curr Biol 2000 10 785 788 10.1016/S0960-9822(00)00566-2 10898980

[B10] Shu HB Johnson H B cell maturation protein is a receptor for the tumor necrosis factor family member TALL-1 Proc Natl Acad Sci USA 2000 97 9156 9161 10.1073/pnas.160213497 10908663 PMC16838

[B11] Thompson JS Bixler SA Qian F Vora K Scott ML Cachero TG Hession C Schneider P Sizing ID Mullen C Strauch K Zafari M Benjamin CD Tschopp J Browning JL Ambrose C BAFF-R, a newly identified TNF receptor that specifically interacts with BAFF Science 2001 293 2108 2111 10.1126/science.1061965 11509692

[B12] Xia XZ Treanor J Senaldi G Khare SD Boone T Kelley M Theill LE Colombero A Solovyev I Lee F McCabe S Elliott R Miner K Hawkins N Guo J Stolina M Yu G Wang J Delaney J Meng SY Boyle WJ Hsu H TACI is a TRAF-interacting receptor for TALL-1, a tumor necrosis factor family member involved in B cell regulation J Exp Med 2000 192 137 143 10.1084/jem.192.1.137 10880535 PMC1887716

[B13] Yan M Marsters SA Grewal IS Wang H Ashkenazi A Dixit VM Identification of a receptor for BLyS demonstrates a crucial role in humoral immunity Nat Immunol 2000 1 37 41 10.1038/76889 10881172

[B14] Yu G Boone T Delaney J Hawkins N Kelley M Ramakrishnan M McCabe S Qiu WR Kornuc M Xia XZ Guo J Stolina M Boyle WJ Sarosi I Hsu H Senaldi G Theill LE APRIL and TALL-I and receptors BCMA and TACI: system for regulating humoral immunity Nat Immunol 2000 1 252 256 10.1038/79802 10973284

[B15] Mukhopadhyay A Ni J Zhai Y Yu GL Aggarwal BB Identification and characterization of a novel cytokine, THANK, a TNF homologue that activates apoptosis, nuclear factor-κB, and c-Jun NH_2_-terminal kinase J Biol Chem 1999 274 15978 15981 10.1074/jbc.274.23.15978 10347144

[B16] Hatzoglou A Roussel J Bourgeade MF Rogier E Madry C Inoue J Devergne O Tsapis A TNF receptor family member BCMA (B cell maturation) associates with TNF receptor-associated factor (TRAF) 1, TRAF2, and TRAF3 and activates NF-κB, elk-1, c-Jun N-terminal kinase, and p38 mitogen-activated protein kinase J Immunol 2000 165 1322 1330 10903733 10.4049/jimmunol.165.3.1322

[B17] Khare SD Sarosi I Xia XZ McCabe S Miner K Solovyev I Hawkins N Kelley M Chang D Van G Ross L Delaney J Wang L Lacey D Boyle WJ Hsu H Severe B cell hyperplasia and autoimmune disease in TALL-1 transgenic mice Proc Natl Acad Sci USA 2000 97 3370 3375 10.1073/pnas.050580697 10716715 PMC16246

[B18] Mackay F Woodcock SA Lawton P Ambrose C Baetscher M Schneider P Tschopp J Browning JL Mice transgenic for BAFF develop lymphocytic disorders along with autoimmune manifestations J Exp Med 1999 190 1697 1710 10.1084/jem.190.11.1697 10587360 PMC2195729

[B19] Groom J Kalled SL Cutler AH Olson C Woodcock SA Schneider P Tschopp J Cachero TG Batten M Wheway J Mauri D Cavill D Gordon TP Mackay CR Mackay F Association of BAFF/BLyS overexpression and altered B cell differentiation with Sjögren's syndrome J Clin Invest 2002 109 59 68 11781351 10.1172/JCI14121 PMC150825

[B20] Jonsson MV Szodoray P Jellestad S Jonsson R Skarstein K Association between circulating levels of the novel TNF family members APRIL and BAFF and lymphoid organization in primary Sjögren's syndrome J Clin Immunol 2005 25 189 201 10.1007/s10875-005-4091-5 15981083

[B21] Mariette X Roux S Zhang J Bengoufa D Lavie F Zhou T Kimberly R The level of BLyS (BAFF) correlates with the titre of autoantibodies in human Sjögren's syndrome Ann Rheum Dis 2003 62 168 171 10.1136/ard.62.2.168 12525388 PMC1754442

[B22] Daridon C Devauchelle V Hutin P Le Berre R Martins-Carvalho C Bendaoud B Dueymes M Saraux A Youinou P Pers JO Aberrant expression of BAFF by B lymphocytes infiltrating the salivary glands of patients with primary Sjögren's syndrome Arthritis Rheum 2007 56 1134 1144 10.1002/art.22458 17393395

[B23] Lavie F Miceli-Richard C Quillard J Roux S Leclerc P Mariette X Expression of BAFF (BLyS) in T cells infiltrating labial salivary glands from patients with Sjögren's syndrome J Pathol 2004 202 496 502 10.1002/path.1533 15095277

[B24] Matsushita T Hasegawa M Yanaba K Kodera M Takehara K Sato S Elevated serum BAFF levels in patients with systemic sclerosis: enhanced BAFF signaling in systemic sclerosis B lymphocytes Arthritis Rheum 2006 54 192 201 10.1002/art.21526 16385515

[B25] Zhang J Roschke V Baker KP Wang Z Alarcon GS Fessler BJ Bastian H Kimberly RP Zhou T Cutting edge: a role for B lymphocyte stimulator in systemic lupus erythematosus J Immunol 2001 166 6 10 11123269 10.4049/jimmunol.166.1.6

[B26] Cheema GS Roschke V Hilbert DM Stohl W Elevated serum B lymphocyte stimulator levels in patients with systemic immune-based rheumatic diseases Arthritis Rheum 2001 44 1313 1319 10.1002/1529-0131(200106)44:6<1313::AID-ART223>3.0.CO;2-S 11407690

[B27] Tishler M Yaron I Shirazi I Yossipov Y Yaron M Increased salivary interleukin-6 levels in patients with primary Sjögren's syndrome Rheumatol Int 1999 18 125 127 10.1007/s002960050070 10220831

[B28] Grisius MM Bermudez DK Fox PC Salivary and serum interleukin 6 in primary Sjögren's syndrome J Rheumatol 1997 24 1089 1091 9195514

[B29] Hirano T Yasukawa K Harada H Taga T Watanabe Y Matsuda T Kashiwamura S Nakajima K Koyama K Iwamatsu A Tsunasawa S Sakiyama F Matsui H Takahara Y Taniguchi T Kishimoto T Complementary DNA for a novel human interleukin (BSF-2) that induces B lymphocytes to produce immunoglobulin Nature 1986 324 73 76 10.1038/324073a0 3491322

[B30] Bauer J Ganter U Geiger T Jacobshagen U Hirano T Matsuda T Kishimoto T Andus T Acs G Gerok W Ciliberto G Regulation of interleukin-6 expression in cultured human blood monocytes and monocyte-derived macrophages Blood 1988 72 1134 1140 3262381

[B31] Tosato G Seamon KB Goldman ND Sehgal PB May LT Washington GC Jones KD Pike SE Monocyte-derived human B-cell growth factor identified as interferon-β2 (BSF-2, IL-6) Science 1988 239 502 504 10.1126/science.2829354 2829354

[B32] Varin MM Le Pottier L Youinou P Saulep D Mackay F Pers JO B-cell tolerance breakdown in Sjögren's syndrome: focus on BAFF Autoimmun Rev 9 604 608 10.1016/j.autrev.2010.05.006 20457281

[B33] Vitali C Bombardieri S Jonsson R Moutsopoulos HM Alexander EL Carsons SE Daniels TE Fox PC Fox RI Kassan SS Pillemer SR Talal N Weisman MH Classification criteria for Sjögren's syndrome: a revised version of the European criteria proposed by the American-European Consensus Group Ann Rheum Dis 2002 61 554 558 10.1136/ard.61.6.554 12006334 PMC1754137

[B34] Le J Vilcek J Lymphokine-mediated activation of human monocytes: neutralization by monoclonal antibody to interferon-γ Cell Immunol 1984 85 278 283 10.1016/0008-8749(84)90299-5 6424948

[B35] Chang SK Arendt BK Darce JR Wu X Jelinek DF A role for BLyS in the activation of innate immune cells Blood 2006 108 2687 2694 10.1182/blood-2005-12-017319 16825497 PMC1895592

[B36] Chang SK Mihalcik SA Jelinek DF B lymphocyte stimulator regulates adaptive immune responses by directly promoting dendritic cell maturation J Immunol 2008 180 7394 7403 18490739 10.4049/jimmunol.180.11.7394 PMC2600490

[B37] Bossen C Schneider P BAFF, APRIL and their receptors: structure, function and signaling Semin Immunol 2006 18 263 275 10.1016/j.smim.2006.04.006 16914324

[B38] Hulkkonen J Pertovaara M Antonen J Pasternack A Hurme M Elevated interleukin-6 plasma levels are regulated by the promoter region polymorphism of the *IL6 *gene in primary Sjögren's syndrome and correlate with the clinical manifestations of the disease Rheumatology (Oxford) 2001 40 656 661 10.1093/rheumatology/40.6.656 11426023

[B39] Huard B Arlettaz L Ambrose C Kindler V Mauri D Roosnek E Tschopp J Schneider P French LE BAFF production by antigen-presenting cells provides T cell co-stimulation Int Immunol 2004 16 467 475 10.1093/intimm/dxh043 14978020

[B40] Lavie F Miceli-Richard C Ittah M Sellam J Gottenberg JE Mariette X B-cell activating factor of the tumour necrosis factor family expression in blood monocytes and T cells from patients with primary Sjögren's syndrome Scand J Immunol 2008 67 185 192 10.1111/j.1365-3083.2007.02049.x 18201372

[B41] Litinskiy MB Nardelli B Hilbert DM He B Schaffer A Casali P Cerutti A DCs induce CD40-independent immunoglobulin class switching through BLyS and APRIL Nat Immunol 2002 3 822 829 12154359 10.1038/ni829 PMC4621779

[B42] Hymowitz SG Patel DR Wallweber HJ Runyon S Yan M Yin J Shriver SK Gordon NC Pan B Skelton NJ Kelley RF Starovasnik MA Structures of APRIL-receptor complexes: like BCMA, TACI employs only a single cysteine-rich domain for high affinity ligand binding J Biol Chem 2005 280 7218 7227 10.1074/jbc.M411714200 15542592

[B43] Cachero TG Schwartz IM Qian F Day ES Bossen C Ingold K Tardivel A Krushinskie D Eldredge J Silvian L Lugovskoy A Farrington GK Strauch K Schneider P Whitty A Formation of virus-like clusters is an intrinsic property of the tumor necrosis factor family member BAFF (B cell activating factor) Biochemistry 2006 45 2006 2013 10.1021/bi051685o 16475789

[B44] Day ES Cachero TG Qian F Sun Y Wen D Pelletier M Hsu YM Whitty A Selectivity of BAFF/BLyS and APRIL for binding to the TNF family receptors BAFFR/BR3 and BCMA Biochemistry 2005 44 1919 1931 10.1021/bi048227k 15697217

[B45] Patel DR Wallweber HJ Yin J Shriver SK Marsters SA Gordon NC Starovasnik MA Kelley RF Engineering an APRIL-specific B cell maturation antigen J Biol Chem 2004 279 16727 16735 10.1074/jbc.M312316200 14764606

[B46] Manoussakis MN Boiu S Korkolopoulou P Kapsogeorgou EK Kavantzas N Ziakas P Patsouris E Moutsopoulos HM Rates of infiltration by macrophages and dendritic cells and expression of interleukin-18 and interleukin-12 in the chronic inflammatory lesions of Sjögren's syndrome: correlation with certain features of immune hyperactivity and factors associated with high risk of lymphoma development Arthritis Rheum 2007 56 3977 3988 10.1002/art.23073 18050195

[B47] Xanthou G Tapinos NI Polihronis M Nezis IP Margaritis LH Moutsopoulos HM CD4 cytotoxic and dendritic cells in the immunopathologic lesion of Sjögren's syndrome Clin Exp Immunol 1999 118 154 163 10.1046/j.1365-2249.1999.01037.x 10540173 PMC1905402

[B48] Gottenberg JE Cagnard N Lucchesi C Letourneur F Mistou S Lazure T Jacques S Ba N Ittah M Lepajolec C Labetoulle M Ardizzone M Sibilia J Fournier C Chiocchia G Mariette X Activation of IFN pathways and plasmacytoid dendritic cell recruitment in target organs of primary Sjögren's syndrome Proc Natl Acad Sci USA 2006 103 2770 2775 10.1073/pnas.0510837103 16477017 PMC1413808

[B49] Zhou LJ Tedder TF CD14+ blood monocytes can differentiate into functionally mature CD83+ dendritic cells Proc Natl Acad Sci USA 1996 93 2588 2592 10.1073/pnas.93.6.2588 8637918 PMC39841

[B50] Wildenberg ME Welzen-Coppens JM van Helden-Meeuwsen CG Bootsma H Vissink A van Rooijen N van de Merwe JP Drexhage HA Versnel MA Increased frequency of CD16+ monocytes and the presence of activated dendritic cells in salivary glands in primary Sjögren syndrome Ann Rheum Dis 2009 68 420 426 10.1136/ard.2008.087874 18397959

[B51] Ozaki Y Amakawa R Ito T Iwai H Tajima K Uehira K Kagawa H Uemura Y Yamashita T Fukuhara S Alteration of peripheral blood dendritic cells in patients with primary Sjögren's syndrome Arthritis Rheum 2001 44 419 431 10.1002/1529-0131(200102)44:2<419::AID-ANR61>3.0.CO;2-U 11229474

[B52] Mitsias DI Tzioufas AG Veiopoulou C Zintzaras E Tassios IK Kogopoulou O Moutsopoulos HM Thyphronitis G The Th1/Th2 cytokine balance changes with the progress of the immunopathological lesion of Sjögren's syndrome Clin Exp Immunol 2002 128 562 568 10.1046/j.1365-2249.2002.01869.x 12067313 PMC1906267

